# Home-Based Monitoring of Eating in Adolescents: A Pilot Study

**DOI:** 10.3390/nu13124354

**Published:** 2021-12-03

**Authors:** Ghassan Idris, Claire Smith, Barbara Galland, Rachael Taylor, Christopher John Robertson, Mauro Farella

**Affiliations:** 1 Metro North Hospital and Health Service, Queensland Children’s Hospital, Brisbane, QLD 4101, Australia; 2 School of Medicine and Dentistry, Griffith University, Brisbane, QLD 4215, Australia; 3 Sir John Walsh Research Institute, University of Otago, Dunedin 9016, New Zealand; drchrisrobertson@gmail.com (C.J.R.); mauro.farella@otago.ac.nz (M.F.); 4 Department of Human Nutrition, University of Otago, Dunedin 9016, New Zealand; claire.smith@otago.ac.nz; 5 Department of Women’s and Children’s Health, University of Otago, Dunedin 9016, New Zealand; barbara.galland@otago.ac.nz; 6 Department of Medicine, University of Otago, Dunedin 9016, New Zealand; rachael.taylor@otago.ac.nz

**Keywords:** eating monitoring, chewing features, adolescents, body mass index, electromyography, automated camera

## Abstract

Objectives: To investigate eating episodes in a group of adolescents in their home-setting using wearable electromyography (EMG) and camera, and to evaluate the agreement between the two devices. Approach: Fifteen adolescents (15.5 ± 1.3 years) had a smartphone-assisted wearable-EMG device attached to the jaw to assess chewing features over one evening. EMG outcomes included chewing pace, time, episode count, and mean power. An automated wearable-camera worn on the chest facing outwards recorded four images/minute. The agreement between the camera and the EMG device in detecting eating episodes was evaluated by calculating specificity, sensitivity, and accuracy. Main results: The features of eating episodes identified by EMG throughout the entire recording time were (mean (SD)); chewing pace 1.64 (0.20) Hz, time 10.5 (10.4) minutes, episodes count 56.8 (39.0), and power 32.1% (4.3). The EMG device identified 5.1 (1.8) eating episodes lasting 27:51 (16:14) minutes whereas the cameras indicated 2.4 (2.1) episodes totaling 14:49 (11:18) minutes, showing that the EMG-identified chewing episodes were not all detected by the camera. However, overall accuracy of eating episodes identified ranged from 0.8 to 0.92. Significance: The combination of wearable EMG and camera is a promising tool to investigate eating behaviors in research and clinical-settings.

## 1. Introduction

Electromyography (EMG) is used to assess masticatory muscle activity and is considered the gold standard method to assess chewing activity. It can accurately identify chewing episodes and their features such as the occurrence time, amplitude, duration, and pace, i.e., the frequency rate of chewing strokes [1]. A potential alternative or a complementary approach to investigate eating behavior is the observation of food consumed from a photo/video recording.

Studying physiological and behavioral aspects of chewing in adolescents has a great importance as they can affect nutritional status [2], are associated with food consumption and may thus impact body weight and health [3,4,5,6,7]. Although chewing features have been widely studied, the majority of this research has been under laboratory conditions, which is a major limitation given the experimental setup may influence the measured masticatory parameters [8]. Furthermore, previous studies have predominantly investigated chewing features in adults using standardized test foods, mostly chewing gum [9,10,11], and have used visual observation or self-report to evaluate the outcome variables [12,13,14,15,16,17,18]. Little information is currently available regarding chewing function in the natural environment, especially in adolescents, and novel techniques are required to objectively evaluate chewing as it naturally occurs outside of laboratory settings.

Automated wearable cameras with wide-angle lenses have the potential to passively record participation in multiple lifestyle behaviors, as well as the environmental context of these different activities, with little respondent burden. Automated cameras have been used to examine a diverse range of health-related behaviors and exposures such as sedentary behavior in adolescents [19,20] and adults [21], physical activity in older adults [22], and food intake in children [23,24] and adults [25,26,27]. 

Cameras may be considered a valuable additional method to aid in evaluating eating episodes in adolescents [28], but no research to date appears to have determined their accuracy in relation to the gold standard EMG method. The use of objective data (EMG and wearable camera) about chewing provides an opportunity to undertake future ecological studies that assess both chewing function and food intake, leading to a better understanding of appropriate support for behavioral treatment approaches for weight control in children [28], and unhealthy eating habits such as a child’s picky eating behavior [29].

The aims of the current pilot study were: (1) to study the features of chewing in a group of adolescents in their home setting utilizing a portable wireless EMG device (a pilot study *n* = 15), and (2) to determine the validity of monitoring eating episodes in a home setting using a wearable camera—in other words, the agreement between the wearable camera and EMG device in detecting eating episodes.

## 2. Materials and Methods

The current study is an observational study that examined the chewing features of a group of volunteer adolescents within their homes. The study was carried out in the University of Otago, New Zealand. A convenience group of volunteers (fifteen adolescents, 13–17 years old) were recruited in New Zealand from Dunedin city, between October 2017 and June 2019. The group comprised 7 females and 8 males. Ethical approval was sought from the University of Otago Human Ethics Committee and was obtained (Ref 17/017). This study was a sub-study of the SNAP IT study (*n* = 160) [20].

Participants were recruited via social media, community networks, notice boards, schools, and word of mouth. The inclusion criteria were defined as (1) age range of 13–17 years; (2) willingness to wear an EMG device and an automated time-lapse camera for one evening. There were no ethnic or gender restrictions. The participants have no condition that affects the chewing behavior. 

On a school day, data were collected during a single home-based study session, starting around 5 p.m. All participants and parents received an instruction booklet explaining detailed information about the study and devices used in the study. After informed consent was obtained, demographic information was collected, and height and weight were measured in duplicate following standard procedures. Body mass index (BMI) z-scores were calculated according to World Health Organization growth standards [30].

**Figure 1:**
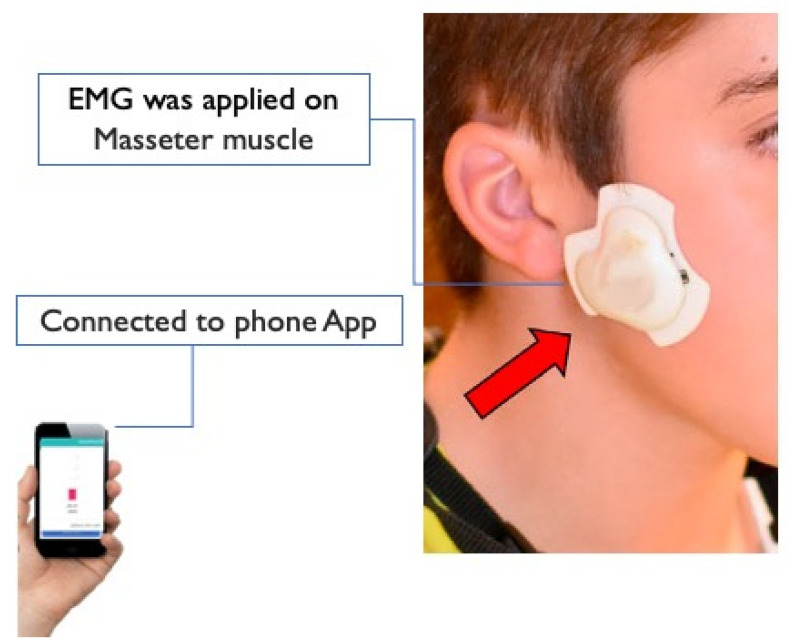
(EMG) wireless device: A small wireless EMG device developed at the University of Otago can be used in natural home-settings to recorder mastication activities connecting to a smart phone using Bluetooth.

A smart phone-assisted wireless EMG device developed by our research team was used to record chewing activity in the home setting without interfering with routine activities or chewing behavior [28,31,32,33]. The EMG device was applied unilaterally on the preferred chewing side of the participant. If no preference was indicated the EMG device was applied on the right-hand side (Figure 1). The electrodes of the EMG device were positioned on the skin over most prominent part of the masseter muscle during contraction and parallel to the main muscle fibers at a center-to-center distance of 20 mm. A third electrode acted as a right-leg drive for noise suppression and was 23.5 mm distal to the active electrodes [33]. A smartphone application (App) was developed in Android to enable visualization, calibration, and logging of EMG activity. The user-friendly App was designed to allow the investigator to set threshold values for the detection of contraction episodes prior to each recording session. The received EMG data were stored in the internal memory of the smartphone [28,33]. 

To normalize the raw EMG activity in the time domain, the maximum voluntary contraction was used as the reference standard. To initiate the maximum voluntary bite effort, a participant was asked to bite into a soft rubber cylinder (Aligner Chewies AC-25GMPP, Dentsply Raintree Essix Glenroe, New Orleans, LA, USA). This rubber cylinder was positioned between the molars on the same side as the EMG recorder and the participant was asked to clench down on the cylinder as hard as possible for 3 s. This process was repeated three times, with a rest time of 30–60 s between each effort. To calibrate the EMG activity in the time-frequency domain, a standardized food was used. The participants were asked to consume three pieces from a small packet of rice crackers (Peckish Cheese Rice Crackers 20 g, Menora, Co., Victoria, Australia); then they were asked to chew gum (Wrigley’s Extra Chewing Gum Spearmint, Mars Incorporated, Mars Wrigley Confectionery, New South Wales, Australia) for one minute. This calibration is essential to calculate the chewing maximum chewing power and to set thresholds for automated identification of chewing activity using our previously developed algorithm [33].

After synchronizing the recording devices via network time, the participants wore the camera secured on their lapel around their neck on a strap sitting on the upper chest and facing outwards to document the environment. The camera (Brinno TLC120-Brinno Inc., Taipei, Taiwan) was worn simultaneously with the EMG device and programmed to take a wide-angled photo every 15 s from 5 p.m. until bedtime.); the camera measured 60 × 60 × 35 mm and weighed 101 g, and could record in low light settings. The participants were also made aware that they could remove the camera or cover it so it would not take photos during operation (for privacy). 

On collection of equipment at the second home visit the participants were able to review the captured images and delete any photos they did not want the researcher viewing. The photos were uploaded from the camera to a laptop, and the participants then reviewed and deleted any images that contained sensitive material before being submitted to the research team.

### 2.1. Data Analysis

After downloading the EMG data recordings from the smartphones, a quality check of the recordings was carried out using an R™ script (R™ software. V3.3.1 R Foundation for Statistical Computing, Vienna, Austria); the recordings were plotted and were used in the subsequent analysis. The downloaded EMG signals were baseband-demodulated with root mean square amplitude values calculated over 125-ms contiguous rectangular windows using the MATLAB software (MATLAB 8.0, MathWorks, Natick, MA, USA). Applying a previously validated algorithm, the demodulated EMG signals were analyzed in the time-frequency domain [1,33,34]. After a quality check, the windowed short-time fast Fourier transform was applied to 64 points with a one-point sliding Hamming window. The resulting spectrogram (spectrum) had a frequency band ranging from 0 to 4 Hz, a frequency resolution of 0.125 Hz, and a time resolution of 125 ms For each spectrum, the peak values of frequency (Hz) and power (dB) were calculated. Power was expressed as a percentage of the maximum recorded power during the standardized chewing tasks. The onset and cessation of chewing episodes were automatically detected through the algorithm based on two thresholds for frequency and percentage of power, set at 0.625 Hz and 10%, respectively. If they were separated by less than two seconds, two chewing episodes were merged into one episode. To obtain an estimate of the frequency and power of a single chewing episode, the peak frequency and percentage of power across each episode’s duration was averaged, and henceforth, in this report, they are simply referred to as frequency and power, respectively. The chewing frequency averaged across a whole recording session was regarded as an indication of an individual’s chewing pace.

**EMG eating episode identification**: A cluster of chewing episodes was considered as an eating episode, where any eating episodes less than 5 min apart were merged into one eating episode. 

**Image analyses**: The images were coded by trained research assistants with nutrition backgrounds using the open-source software TimeLapse2 (http://saul.cpsc.ucalgary.ca/timelapse/, accessed on 17 August 2021). This software provides users with a customizable interface to code images (or videos). The protocol for coding eating episodes and the context of eating episodes was developed by three researchers. Camera images were annotated with respect to specific foods and beverages eaten and the context of each eating occasion (where and with whom) in two steps. First, all images with any food or eating-related activity were identified. This could include images where the participant was in the kitchen or sitting at a table with food. Second, images showing clear evidence of the participant eating such as: eating utensils; movement of hand to mouth; plates, bowls, and glasses with diminishing amounts of food were identified. These images were coded for (i) eating occasion (meal or snack); (ii) where the participant was eating; (iii) the number of person(s) present; and (iv) allocated food code(s). Each food visible in the image was assigned a food code. The food codes were adapted from the 2008/09 New Zealand Adult Nutrition Survey food coding classification. The food coding scheme contains 540 unique codes organized into 120 sub-groups, which can be further collapsed into 35 main food groups. This coding system was used in the wider SNAP IT and a coding manual and protocol was developed to ensure coding decisions were consistent. The images were coded by three researchers with nutrition backgrounds. In the current study, we used the available data about timing and duration of consumed food to identify the eating episodes. 

**Camera eating episode identification**: An eating episode was defined as a continuous period of eating. Any images with evidence of eating within five minutes of the last image of a continuous series were defined within the same eating episode, to match the time frames used in the EMG episode identification. Images of food that were identified greater than five minutes from the last image containing food were defined as a new eating episode. 

### 2.2. Validation Procedure and Analysis

A quality check was performed to assess the quality of the recorded EMG signal and the camera images acquired by the participants. Time synchronization of the EMG device and camera was then confirmed.

Start and end times of the EMG and camera recordings were derived from the camera start/end times, since the camera was started slightly later than the EMG device and was stopped earlier by the participants. The start/end time was used to cut the EMG recording. This ensured that comparable EMG and camera data were obtained for each participant.

We aimed to test the agreement between the eating episodes detected by the camera and those detected by the EMG device, we considered the EMG device to be the gold standard, as it has been previously validated [34]. Accordingly, a true positive was defined as when both recordings (EMG and wearable camera) detected eating activity, a true negative when both recordings had no eating activity detected (eating-free time), a false negative when the EMG device detected eating activity and the camera did not, and a false positive when the camera detected eating activity and no detection was observed on the EMG.

Accuracy, specificity, and sensitivity were defined as follows: accuracy represents the proportion of true positive and true negatives eating episodes detected by the camera in relation to the eating episodes detected by the EMG device; sensitivity represents the true positive rate, which is the proportion of eating episodes that are correctly identified by the camera in comparison to the EMG-detected eating episodes; specificity represents the true negative rate which is the proportion of eating-free times that are correctly identified by the camera in comparison to the EMG-identified eating-free times. 

### 2.3. Statistical Analysis

**Figure 2:**
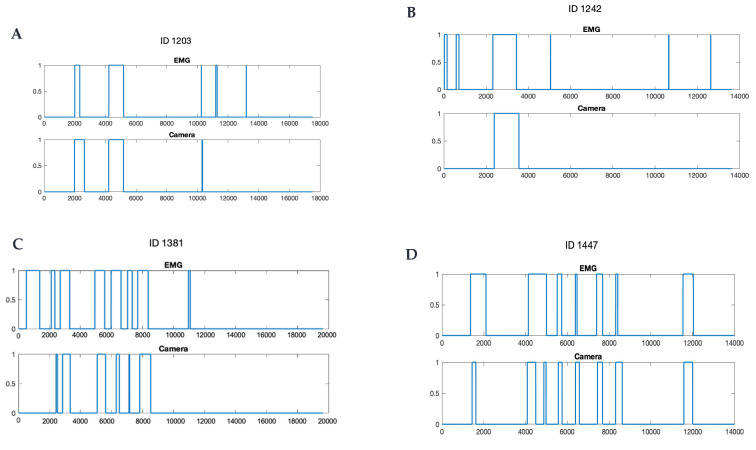
Examples of chewing episodes detections by the electromyography (EMG) and the camera in timeline (seconds). Note that the EMG device generally detected more eating episodes (**A**–**C**) and a shorter eating time, even when the number of episodes identified was the same (**D**). 0 represents no eating activity, 1 represents eating activity.

The statistical analyses were performed using SAS (version 9, SAS Institute, Cary, NC, USA). Firstly, conventional descriptive statistics were performed. Variability was expressed as a standard error of the mean (±SEM); median, first and third quartile were reported. The agreement between eating episodes detected by the camera and the EMG was determined by calculating accuracy, specificity, and sensitivity using two different analytical approaches: (1) episode-wise analysis, which assessed the agreement between discrete eating episodes detected by the two methods (EMG vs. camera); and (2) timepoint-wise analysis, which assessed the agreement between the two methods along the time line in every one second in the EMG recording and in every 15 s in the camera data. Examples of the timepoint-wise comparisons are illustrated in Figure 2.

## 3. Results

Demographic information is summarized in Table 1. Most of the participants indicated that the right side was their preferred chewing side (*n* = 11), with the remainder preferring the left side (*n* = 4). 

Chewing features as evaluated by EMG recordings are summarized in Table 2. EMG recording analysis showed an average chewing pace of 1.64 Hz ± 0.20 Hz, a chewing power of 32.1 ± 4.3%, average chewing episodes count of 56.8 ± 39.0, and an average chewing time of 10.5 min ± 10.4 min for the course of their recorded evening eating occasions. The EMG device identified 5.1 (1.8) eating episodes lasting 27:51 (16:14) minutes, whereas the cameras indicated 2.4 (2.1) episodes totaling 14:49 (11:18) minutes, showing that the EMG-identified chewing episodes were not all detected by the camera. These data highlight that the camera misses a significant portion of the eating episodes identified through the EMG device. However, the main evening meal was correctly identified by both the camera and the EMG device in 14/15 participants, with an accuracy of 0.93. In one participant, the camera was not able to detect the main dinner meal.

Table 3 summarizes the descriptive statistics for the number of eating episodes detected by the EMG device and the camera; Figure 2 shows graphs of four examples of the comparisons between the camera- and the EMG-detected eating episodes in the timeline. 

In general, the accuracy of the camera in detecting the eating episodes using the timepoint-wise analysis ranged from 0.80 to 0.98. Sensitivity was estimated to be 0.34 and ranged from 0.00 to 0.92, whereas specificity was estimated to be 0.99 and ranged from 0.94 to 1.00. 

## 4. Discussion

This pilot study demonstrated that a wearable camera has reasonable accuracy for determining the number of evening eating episodes in adolescents in their natural home setting. While the camera did record a lower number of episodes than that of the EMG device as indicated by a relatively low sensitivity, specificity was extremely high (indicating clearly when the participant was not eating), resulting in an overall accuracy greater than 80%. 

A wearable, wireless EMG device was used in the current study. EMG devices are widely used to assess masticatory muscle contractions as they provide an objective, valid, and reproducible method of recording muscle contractions [35,36]. EMG recording devices were first introduced for stationary use, and only later adapted to be wireless and therefore portable. In the current study, the development of a wearable, wireless EMG device to record masticatory muscle activity and analyze oral behaviors, came after several attempts [37]. This device has overcome many of the drawbacks of previous lab-based work, which were only able to focus on sleep-time EMG activity, mainly aiming to study nocturnal bruxism [38,39,40]. They could not provide comprehensive analyses of daytime EMG evaluation for a long duration while carrying out routine activities. Moreover, laboratory experiments are unlikely to represent the natural environment which may affect oral and chewing behaviors. The development of wearable and wireless EMG [32] is timely, given the increasing interest in recording masticatory muscle activity and oral behaviors during the daytime and in natural settings.

In previous studies, manual scoring of video or EMG recordings, has been widely used for the assessment of mastication activities. The manual/visual scoring of jaw movements relies on the subjective evaluation and skill of the examiner. This method is expensive and time-consuming. To overcome the limitations of such methods, an automated method of assessing rhythmic masticatory muscle activity (RMMA) using EMG recordings has been developed and used in the current study. The algorithm automatically detects the onset and cessation of chewing episodes. As well as the number of episodes, it also assesses chewing pace (frequency), amplitude, and duration, without examiner interaction. The algorithm can accurately differentiate chewing from head movements, speaking, yawning, and clenching teeth, which reduces the chance of false positives [34]. This was clearly demonstrated by our data, which showing a false positive rate of 1.5%.

The present research focused on indirect, continuous recordings of chewing episodes in free-living individuals. Instead of targeting macro-nutritional composition or total calorie intake (what and how much is eaten), our approach focused on the occurrence of eating episodes (when, how long, what pace) indicated by EMG analysis of chewing activity. Identifying meal timing has an increased importance when studying eating behaviors as it may be a critical modulator of health outcomes due to complex interactions between circadian biology, nutrition and human metabolism [41].

The reasoning is that any fully automatic classification of food content and amount is currently very difficult and imprecise. The alternative to automatic classification of food intake is self-report. A major problem with this method is under-reporting, which may be due to unconscious omission of eating occasions, recording fatigue, or conscious misreporting [42]. Furthermore, it has been found that between-meal snacks, especially unhealthy snacks, can be frequently omitted from participants’ self-report, with more than one third of snack consumption being unaccounted for; however, the main meals are well reported [43,44]. 

An automated wearable camera with a wide-angle lens was used in the current study. Wearable cameras are an emerging technique to passively capture multiple lifestyle behaviors and the surrounding context of these behaviors with minimal respondent burden. Although, a relatively new research tool, automated cameras have been used to examine a diverse range of health-related behaviors and exposures, including: sedentary behavior in young [18] and older adults [21], physical activity in older adults [22], diet in adults [25,26] and children [23], food purchasing in adolescents whilst commuting [45], exposure to advertising in children [46,47,48,49] and television viewing in adults [50].

Images taken with handheld devices or wearable cameras have been used by dieticians when assessing diet and estimating portion size (image-assisted methods). Image-assisted approaches can supplement either dietary records or 24 h dietary recalls [25,26,51]. In recent years, image-based approaches integrating application technology for mobile devices have been developed (image-based methods). Image-based approaches aim to capture all eating occasions with images being the primary record of dietary intake. The captured images are the main source of information and only use additional input from the user as verification. The image capturing can be passive, meaning that at a defined time-frequency, the device automatically takes an image, whereas active approaches require the participant to take images manually. The current literature suggests that image-assisted methods can improve the accuracy of conventional dietary assessment methods by adding eating occasion details via pictures captured by an individual (dynamic images). Under-reporting is reduced when using dynamic images compared with traditional assessment methods [52,53,54]. In the current study, we used the captured images solely to identify the eating occasions (eating episodes), rather than to estimate calorie intake, which is a significant shift from previous diet assessment studies. 

The camera showed a low sensitivity alongside its high specificity. This may have occurred as a result of, particularly at snack times, only capturing four images every minute which may not have been frequent enough to detect short snacking activities. However, the camera was still able to detect the presence of an evening meal in almost all the participants. Through analyzing the camera data of 15 participants, the camera did not provide enough information in one participant to detect the evening meal as that participant was not eating the meal in a dining room, and was trying to avoid the camera when eating, which resulted in a very few photos showing food while eating. Therefore, this meal was missed when the images were coded.

Using a camera alone to assess the number of eating episodes may result in under-detection of snacking episodes; this is also, the main issue observed with self-reporting. Using an automated camera setting of capturing frequency greater than 4/min snapshots may allow for more accuracy in detecting short snacking and eating episodes if the camera is used alone. The combined use of both the camera and the EMG device appears to be a promising technique for an improved assessment of eating behaviors. The EMG accurately collects chewing information and the camera provides details about the consumed food and the environmental context of the eating behaviors (eating alone/with group, watching TV, etc.). 

Overall, although our combined (EMG and camera) technique in the current study ignores specific food content and amount, it captures important eating episode characteristics. Both devices capture time and duration of each eating episode, additionally, the EMG device measures the amplitude and frequency of chewing strokes. One of the fundamental advantages of the method used in this study, is that the automation of the devices eliminated a significant amount of participant burden. Previous studies have demonstrated inaccuracies related to identifying eating occasions, identifying the food type, and labelling the images, when carried out manually by participants. The key to co-operation is to simplify the process (turning on the camera and the EMG device) and place the burden on technology rather than the user, which we aimed to achieve in the current study. Combined use of a camera with the EMG can provide additional information including the type of food consumed and context of consumption. Using an EMG device with a camera could quicken the image coding by enabling the researcher to filter images with chewing time-points detected by the EMG.

## 5. Strengths of the Study

The current study used EMG as an objective method to validate the use of the camera to detect eating episodes (EMG is considered the gold standard). Recording of mastication in natural home-settings avoided the flaws associated with lab-observation methods. Unlike previous studies that included self-reporting, the current study combined camera recordings with an objective detection of eating episodes (i.e., EMG) Moreover, the EMG device used in the study was unobtrusive as it was small in size, and wireless. 

## 6. Study Limitations

Using cameras in home settings may pose a privacy issue. Moreover, there is a possibility that observation may affect = eating behavior in some adolescents as they know that what they are eating will be recorded. Considering the pilot nature of this study, the results need to be confirmed with a larger group of participants. The current study did not quantify the participants’ satisfaction with acceptability of wearing the EMG device and the camera, a point that may be tested in a future qualitative study.

The sampling frequency of the automated camera was set at 4 images/min. The current study did not test different sampling frequencies to check that an image every 15 s is as accurate as higher capturing frequency to assess eating episodes. This can be tested in a future study. EMG does not record dietary intake, and we did not include analysis of the consumed food (food type, healthy/ unhealthy, etc.).

## 7. Future Directions

Based on the findings of the current study, the combination of an EMG device and a wearable camera can be a useful research tool for accurately studying eating behaviors. Moreover, a smart phone App can be developed to collect and analyze the chewing data in real time. The output of the chewing analysis can be shown on-screen using the App, potentially using the system as a bio-feedback tool to support behavior therapy for childhood obesity. Camera images can be analyzed to track the type of food consumed during the day and other activities associated with eating behavior (who with, where eaten, what context).

A future modification to this system could be to incorporate an EMG-based device, capable of detecting chewing episodes, that subsequently triggers a wearable camera to turn on and capture eating episodes. This could effectively eliminate at least the unconscious sources of under-reporting and heavily reduce memory/recall biases. Such ‘omission free’ reporting of food intake in natural settings could advance basic eating behavior research and inspire new awareness-based eating behavior interventions.

## Figures and Tables

**Table 1:** Demographic and clinical data of the study participants (*n* = 15).

Variable	Mean	SD	Min–Max
Age (years)	15.5	1.3	13.6–17.6
BMI (kg/m^2^)	23.1	4.6	17.8–33.6
BMI (z score)	0.73	1.06	−0.95–2.94
BMI distribution	Normal	Overweight	Obese
*N* (%)	8 (53.3)	5 (33.3)	2 (13.3)
Sex distribution	Female	Male	
*N* (%)	7 (46.7)	8 (53.3)	

BMI (Body Mass Index).

**Table 2:** Chewing features as determined by EMG analysis.

Measure	Mean	SD	SE	25th Pctile	Median	75th Pctile	Min	Max
Chewing pace (Hz)	1.64	0.2	0.1	1.5	1.7	1.9	1.3	2.07
Chewing power (%)	32.1	4.3	1.1	21.8	29.9	40.1	23.9	40.4
Chewing episodes count (*n*)	56.8	39.0	10.1	33.0	52.0	61.0	15.0	185.0
Chewing time (min)	10.5	10.4	2.7	4.0	7.3	12.8	1.6	40.1

SD (Standard Deviation), SE (Standard Error), Min (Minimum), Max (Maximum), 25th Pctile (First Quartile), 75th Pctile (Third Quartile).

**Table 3:** Descriptive statistics for the number of eating episodes detected by the EMG device and the camera.

Participants	Eating Episodes (EMG)						Eating Episodes (Camera)					
	Mean	SD	25th Pctile	Median	75th Pctile	Min–Max	Mean	SD	25th Pctile	Median	75th Pctile	Min–Max
Number	5.4	1.8	4.75	5	6.25	2–9	2.4	2.1	1	1	3.25	1–8
Total eating time (min: s)	27:51	16:14	16:27	23:19	40:00	4:35–67:38	14:49	11:18	01:30	12:45	23:41	00:30–34:15

EMG-detected eating episode: The algorithm allowed for the automated detection of onset and cessation of chewing episodes based on 2 thresholds for frequency. When 2 chewing episodes were separated by less than 2 s, they were merged into 1 episode. A cluster of chewing episodes that were considered as a single eating episode and any two eating episodes with a stand-by time less than 5 min were merged into one eating episode. Camera detected eating episode: Start of an eating episode was identified whenever an image was identified as an eating activity and stopped when that activity ceased. The standby time to separate between two different eating episodes was set at 5 min. SD (Standard Deviation), SE (Standard Error), Min (Minimum), Max (Maximum), 25th Pctile (First Quartile), 75th Pctile (Third Quartile).
